# The Impact of Inflammation on the Immune Responses to Transplantation: Tolerance or Rejection?

**DOI:** 10.3389/fimmu.2021.667834

**Published:** 2021-11-22

**Authors:** Mepur H. Ravindranath, Fatiha El Hilali, Edward J. Filippone

**Affiliations:** ^1^ Department of Hematology and Oncology, Children’s Hospital, Los Angeles, CA, United States; ^2^ Terasaki Foundation Laboratory, Santa Monica, CA, United States; ^3^ Mohamed V Hospital, Moulay Ismail University, Meknes, Morocco; ^4^ Division of Nephrology, Department of Medicine, Sidney Kimmel Medical College at Thomas Jefferson University, Philadelphia, PA, United States

**Keywords:** inflammation, tolerance, rejection, allograft, endothelial cells, exosomes, IL-6

## Abstract

Transplantation (Tx) remains the optimal therapy for end-stage disease (ESD) of various solid organs. Although alloimmune events remain the leading cause of long-term allograft loss, many patients develop innate and adaptive immune responses leading to graft tolerance. The focus of this review is to provide an overview of selected aspects of the effects of inflammation on this delicate balance following solid organ transplantation. Initially, we discuss the inflammatory mediators detectable in an ESD patient. Then, the specific inflammatory mediators found post-Tx are elucidated. We examine the reciprocal relationship between donor-derived passenger leukocytes (PLs) and those of the recipient, with additional emphasis on extracellular vesicles, specifically exosomes, and we examine their role in determining the balance between tolerance and rejection. The concept of recipient antigen-presenting cell “cross-dressing” by donor exosomes is detailed. Immunological consequences of the changes undergone by cell surface antigens, including HLA molecules in donor and host immune cells activated by proinflammatory cytokines, are examined. Inflammation-mediated donor endothelial cell (EC) activation is discussed along with the effect of donor-recipient EC chimerism. Finally, as an example of a specific inflammatory mediator, a detailed analysis is provided on the dynamic role of Interleukin-6 (IL-6) and its receptor post-Tx, especially given the potential for therapeutic interdiction of this axis with monoclonal antibodies. We aim to provide a holistic as well as a reductionist perspective of the inflammation-impacted immune events that precede and follow Tx. The objective is to differentiate tolerogenic inflammation from that enhancing rejection, for potential therapeutic modifications. (*Words 247).*

## Introduction

“Of all the obstacles that must be surmounted to achieve the successful Tx of tissue and organs from one human being to another, the immunological one is the most formidable” - Billingham RE, Barker CF 1969 (1)

Transplantation (Tx) remains the optimal therapy for the end-stage disease (ESD) of various solid organs, including kidney, liver, heart, and lung. The ESD itself as well as the subsequent Tx severely impact the cellular and humoral components of both innate and adaptive immunity. Despite improved short-term graft survival, chronic alloimmune injury remains the major cause of long-term allograft loss. However, some patients achieve tolerance of their grafts, under current immunomodulatory protocols, even after discontinuation of all immunosuppression. The immunodynamics of transplantation, herein defined as dynamic changes in the immune system following implantation, are a prime determinant of this balance between tolerance and rejection.

The purpose of this review is to highlight the important role that inflammation plays in the immunodynamics of transplantation. We will discuss in detail pre-transplantation recipient inflammation and the mediators involved. An in-depth analysis of recipient cells invading an allograft following implantation will be provided, and their roles in rejection and tolerance will be highlighted. We note the reciprocal relationship between donor-derived passenger leukocytes (PLs) and the recipient’s immune system, and we highlight the prime importance of donor-derived extracellular vesicles (EVs), including exosomes. The concept of recipient antigen presenting cell (APC) “cross-dressing” will be discussed in detail. Donor endothelial cell (EC) activation will also be discussed along with the effect of donor-recipient EC chimerism. Finally, as an example of a specific inflammatory mediator, a detailed analysis will be provided regarding the role of IL-6 and its receptor on the immunodynamics of transplantation. Our objective is to provide a holistic as well as a reductionist perspective of the inflammation-associated immune events that precede and follow transplantation. The hope is that tolerogenic-inducing inflammation can be differentiated from that enhancing rejection with potential therapeutic modifications. Many questions remain unanswered, and these will be highlighted throughout.

## Pre-Transplant Inflammation and Immune Mediators in Both Recipients and Donor Organs

End-stage organ disease per se induces pretransplant inflammation in transplant recipients. Patients with pre-existing HLA sensitization may receive immunosuppressive desensitization therapies to lower the level of circulating HLA allo-antibodies. These protocols may include plasma exchange, intravenous immunoglobulins, and depleting antibodies such as rituximab (B-cell), thymoglobulin (B and T cell) and alemtuzumab. The immunosuppressive therapies could promote infections and pro-inflammatory factors (**Table 1**) (2–7). These proinflammatory stimuli may differ from patient to patient, depending on the organ transplanted, the specific infection, therapies administered, and the genetic factors. Specific biomarkers that have been identified post-Tx include C-reactive protein (CRP), hypoalbuminemia, Glasgow Prognostic Score, neutrophil count (PNC), macrophage (MP), neutrophil-lymphocyte ratio (NLR), platelet-lymphocyte ratio (PLR), systemic immune-inflammation index (SII), and proinflammatory cytokines (e.g., IL-1α, IL-Iβ, IL-6, TNFα) (8–10). For example, inflammation during chronic kidney failure (11, 12) impacts both immune responses with the accumulation of monocyte–MPs (13) and the production of pro-inflammatory cytokines. The CRP (14) and anti-αGalactosyl antibody (15) levels correlate with failure of hemodialysis.

**Table 1:** Infectious agents emerging after administration of Immunosuppressive agents in Transplant patients.

Immuno suppresive agents administered in transplant patients	The Purpose of the immunosuppressive agents	Infectious agents emerging consequent to the specific immunosuppression
*Rituximab*	B-cell depletion	Hepatitis B
		Respiratory Viruses
		Gastrointestinal Infection
*Alemtuzumab*	B-cell-T-cell interactions	Bacteria
		Fungi
		Protozoa
		Herpes
		Cytomegalovirus
		Pneumocystis jirovecci
*Etanercept*	TNF inhibition	Bacteria, Tuberculosis, Hepatitis B
*Adalimumab*		
*Infliximab*		
*Gemtuzumab*	CD 33 inhibition	Bacteria,Fungi
*Tocilizumon*	IL-6 inhibition	Bacteria,Clostridium difficile
*Acetemro*		Fusarium, Candida

Additionally, organs from either alive or deceased donors are implanted with varying levels of preexisting donor-derived inflammation. Inflammation-associated immune mediators are often organ and tissue-specific, which include cells and subcellular fractions, including circulatory and urinary microvesicles and exosomes, molecular species (proteins, glycan, and lipoidal), and even sub-molecular fractions, including DNA fragments and miRNA (16–22). These mediators exist even before implantation.

The organs from donation after circulatory death (DCD) and from extended criteria donors (ECDs) are more susceptible to inflammation from ischemic-reperfusion injury (IRI) compared to living donors. Consequently, there is an increased risk of primary non-function and delayed graft function (23–25). In deceased donors, brain death induces a cytokine storm (IL-6 and MCP-1) that results in inflammation, leukocyte infiltration, complement system activation and oxidative stress. Microglial cells get activated and augment the production of cytokines, glutamate, proteases, lipids, polyunsaturated fatty acids, and their metabolites.

The recipient characteristics that impact the degree of inflammation include age, sex (parous or non-parous, if females), and prior sensitization events (transfusion, pregnancy, and failed previous allografts). Additionally, nonspecific events may contribute to activation of the recipient immune system, such as viral, bacterial, or fungal infections. Other factors include pre-existing disease conditions (diabetes, autoimmune diseases, and hypertension) and the type of medication and their doses used on pre-transplantation, including immunosuppressive drugs. Taking into consideration of all the above-mentioned factors, a holistic approach is important for maximizing the allograft survival in an individual recipient. Selected aspects are now discussed in detail.

## Inflammation and Immune Events Associated With Implantation Surgery

Open surgery *per se* (26) induces inflammation by activating cellular mediators of both innate (CD56+ NK-cells, CD14+ monocytes, extravillous trophoblasts, monocytes, and immature dendritic cells) and adaptive (T and B lymphocytes) immunity. The nature and propensity of the inflammatory mediators upon implantation vary with the type of organ transplanted (e.g. kidney, liver, heart, intestine, lungs, and pancreas). Most importantly, the activation of donor cells leads to changes in the profile of surface proteins, such as the upregulation of monomeric α-heavy chains (α-HCs) of HLA class I molecules that are devoid of β-chain (27–30). The cytoplasmic tail of these α-HCs may get tyrosine phosphorylated and can be involved in signal transduction (31). Subsequently, matrix membrane proteases dissociate and release soluble HLA. The recipients’ humoral and cellular immune components interact with both membrane-bound and soluble HLA. These monomeric variants of HLA, also known as open conformers, are highly immunogenic for they expose epitopes cryptic on intact HLA (32).

An acute inflammatory response is capable of abruptly destroying an allograft within minutes after implantation, so-called hyperacute rejection, a major cause of primary non-function. The rate of primary non-function of first deceased donor kidney grafts was 8% in 7788 first grafts, 14% in 1471 second grafts, and 20% in 224 third grafts (33), illustrating the inflammation-associated heightened immune response of re-Tx. One report (34) identified 56 cases of early renal graft loss (never recovered renal function and/or graft thrombosis <48 h after Tx). Fourteen cases were caused by immune-mediated vascular blockage leading to acute vascular rejection. In another report, antibody-mediated, hyperacute vascular rejection was observed soon after liver Tx (35), particularly in individuals with preformed allo-Abs against both HLA and non-HLA antigens.

## Allograft Inflammation Activates and Stimulates Infiltration of Immune Cells

While inflammation pre-exists in a patient with end stage disease undergoing transplant surgery, the transplanted organ or tissue will further stimulate both innate and adaptive immune responses. Inflammation initiates bi-directional movement of donor and recipient immune cells (ICs) (35–39).

Based on the severity of the inflammation in both the graft itself and the host microenvironment, the graft is subjected to an acute or chronic shock response. Different pathways of innate and adaptive immunity-based shock induction have been suggested for different solid organs (35–39). The recipient’s ICs may surround the graft endothelial cells (ECs) and recognize the unique and unfamiliar antigens, which constitute the “primary immunogens”. The donor-ICs migrating from the transplanted organ to the recipient’s regional lymph nodes expose incompatible antigens. In the direct pathway, donor-APCs expressing donor intact HLA molecules are recognized by recipient effector cells. In the indirect pathway, shed donor-HLA molecules are taken up and processed by recipient APCs and presented to recipient lymphocytes as peptides in recipient HLA molecules. In the semidirect pathway, recipient APCs may take up and express intact donor HLA molecules. These allorecognition mechanisms are elaborated below.

### Circulating Immune Cells Infiltrating Renal Allografts Correlate With Rejection

Strom and co-investigators (40–45) studied viable cells recovered from 10 rejected human renal allografts. An abundant and heterogeneous population of cells including MPs and both T and B cells were observed. The isolated live infiltrating lymphocytes (B or T) from the recipient exerted a specific cytolytic effect on ^51^Cr-labeled peripheral blood lymphocytes (B or T) bearing donor antigens. This effect ranged from 7 to 44% in nine of 10 cases. Cytolysis was closely correlated (r = 0.91, p < 0.05) with the histologic grade of cellular rejection but not with humoral rejection, suggesting the role of cytotoxic T cells (CTLs) in T-cell mediated rejection (TCMR). Further examinations by limited fractionation techniques revealed that both T cells and non-T cells (monocyte – MPs) that expressed Fc receptors are responsible for the cytotoxicity. Since about 50% of cells recovered bore Fc receptors, the rejection was suggested to involve antibody-dependent cellular cytotoxicity (ADCC). The CTLs are antigen-specific, and MHC-restricted T cells were shown to infiltrate rejecting allografts.

Sablik et al. (46) showed that T cells and MPs were the dominant cell types in the glomeruli of kidney allograft recipients with chronic active antibody-mediated rejection (ABMR). CD8+ T cells comprised 62% of CD3-positive cells and 68% of MPs were CD68+/CD163+. The tubulointerstitial (TI) compartment showed a mean of 116 CD3+ cells/HPF, of which 54% were CD8+T cells. The MP count in the TI was 21.5 cells/HPF with 39% CD68+ CD163+. B cells (CD20+) were occasionally detected in glomeruli, whereas B-cell aggregates were frequently observed in the TI compartment. Natural killer cells were found in *low* numbers. Surprisingly, increased T regulatory cells (CD3+FoxP3+ T-regs) in the TI compartment were correlated with a decreased, not increased as noted in liver allografts (*vide infra*), survival rate (*p* = 0.004).

Carpio et al. (47) evaluated B-cell expression patterns and association with function and survival in dysfunctional kidney allografts. The patients were evaluated in 3 groups according to the Banff classification: no rejection (40 patients), TCMR (50 patients), and ABMR (20 patients). The CD138-positive plasma cell-rich infiltrates predominated in ABMR and were associated with both stronger reactivity against panel antibodies (r = 0.41; P ≤.001) and the presence of donor-specific antibodies (DSA) (r = 0.32; P ≤.006). The CD20-positive lymphocytes were associated with TCMR, increased HLA mismatch, and the frequency of reTx. The CD138-positive cell infiltrates also were significantly greater in patients who had late rather than early rejection. In multivariate analysis, C4d staining was the only risk factor associated with graft loss.

Recently, Filippone and Farber (48) reviewed the implications of B lineage cells (CD20+ B cells and CD138+ plasma cells) in kidney allografts. B-cells tend to form nodules which may evolve into tertiary lymphoid organs, whereas plasma cells are distributed more diffusely throughout the interstitium in affected cases. B-cell clusters have been associated with steroid-resistant rejection and reduced graft survival in some studies but not in others, and plasma cells may be associated with either TCMR and/or ABMR (48). Whereas both cell types may contribute to allograft injury through antibody production, antigen presentation to T-cells, and cytokine secretion, both cell types may also be tolerogenic. “Given the ability to target B-cells with anti-CD20 monoclonal antibodies and plasma cells with proteasome inhibitors and anti-CD38 monoclonal antibodies, it is increasingly important to determine the significance of such infiltrates” (48).

Reitamo et al. (49) and others (50–54) have observed that monocytes (MO) were the predominant cells in peritubular and glomerular capillaries, while T lymphocytes were localized primarily in perivascular and periglomerular areas. In comparing the localization of different ICs among different biopsies of kidney allografts, a severe acute rejection occurred in association with a marked MO infiltration representing more than 50% of the inflammatory cells (53). However, another study (50) failed to observe a statistical correlation between infiltration of glomerular MOs and allograft outcome. Hancock et al. (51) found the presence of a relatively large number of interstitial MOs during acute rejection (38%-60% of infiltrating leukocytes), and the largest percentage was observed in severe acute rejection cases. In chronic rejection, the MO to T-cell ratio in glomerular and peritubular capillaries (PTC) was significantly increased in kidneys with C4d deposition in the same sites, supporting a role for MOs in ABMR. MOs have also been detected in the thickened intima of arteries with chronic transplant arteriopathy (a manifestation of chronic rejection) (52, 53). These MOs show increased expression of PDGF-B. This factor stimulates migration and proliferation of smooth muscle cells, suggesting a role for MOs in the development of transplant arteriopathy (54).

Halloran and co-investigators (55–67) studied extensively the molecular phenotype of the cell types in biopsies from different allografts (renal, liver, heart, and lungs) to document and reconfirm the diversified roles of different ICs in the immunodynamics of Tx. They found several types of ICs involved, including T and B lymphocytes, NK cells, MPs, and dendritic cells (DCs). In a cohort of renal transplant patients, six C4d-positive ABMRs, six C4d-negative ABMRs, and six TCMRs were found. Analyzing biopsies for CD3, CD68, and CD56 cell markers, they found that the average number of CD56+ NK cells (p=0.006) and CD68+ MPs (p=0.03) in peritubular capillaries was higher in C4d-positive or C4d-negative ABMR biopsies *versus* those with TCMR. There was not such a marked difference with CD3+T cells (p=0.09). Hirohashi et al. (67) observed that DSAs mediate chronic allograft vasculopathy in murine heart allografts through NK cells by an Fc-dependent manner.

### Circulating Immune Cells Infiltrating Liver Allografts Correlate With Tolerance

In liver allografts different kinds of infiltrating ICs may promote tolerance as opposed to rejection. Hepatic infiltrates in operationally tolerant patients show enrichment of regulatory T cells (T-regs) before proinflammatory genes are downregulated (68). Monitoring the frequency of T-reg and Foxp3 mRNA expression among peripheral blood MOs in 12 hepatic allograft recipients undergoing withdrawal of immunosuppression, a progressive increase in circulating CD4+CD25+Foxp3+ T-reg and Foxp3 mRNA expression was observed (69, 70). The expression of adenosine deaminase, which degrades adenosine to evoke stronger T-reg activation, was higher in five tolerant allograft recipients compared to 12 non-tolerant recipients, suggesting that the expression of this enzyme may predict tolerance of liver transplants (71). In addition to T-regs, in peripheral blood of seven operational tolerant pediatric recipients and eight pediatric recipients on low dose immunosuppression, a specific T cell subset (CD4+CD5+CD25+CD38−/lowCD45RA−), correlated with liver allograft tolerance. This specific CD5+ T cell subset is crucial in promoting T-reg induction (72).

Similarly, comparing 19 liver allograft recipients on immunosuppression, including some operationally tolerant patients, with 24 age-matched healthy volunteers, it was noted that the ratios of T-regs/Th17, Th1/Th17, and CD8+/Th17 cells were increased in tolerant patients compared with non-tolerant patients during immunosuppression tapering. The elevated T-regs/Th17 ratio continued over 60 months of follow up in tolerant patients, indicating a reciprocal balance between T-regs and Th17 that may contribute to the development and maintenance of tolerance (73). In another study, 13 tolerant pediatric hepatic allograft recipients showed an elevated ratio of plasmacytoid-DCs (pDC) to myeloid-DCs (mDC) compared to 12 patients remaining on immunosuppression. Notably, a high PDL1/CD86 ratio on pDC correlated with increased T-regs and correlated with pediatric liver allograft tolerance (74). Recently, Dai et al. (75) reviewed “spontaneous” liver transplant tolerance in humans, focusing on the clinically significant role played by T-regs in liver allografts after immunosuppression withdrawal. These investigations emphasized the need to assess T-regs/Th17 ratios pre- and post-Tx in other organ transplants. Thus, there are significant differences in the cell types invading one allograft compared to another, perhaps depending on factors such as type of organ, duration of allograft, type of rejection (acute *versus* chronic), immunologic risk (degree of mismatch, DSA), and prevalence of different inflammatory biomarkers in the allograft microenvironment and within the allograft per se.

## Allograft-Associated Immune Cells Respond to Inflammation: Double Immune Reaction, Host-*Versus*-Graft, and Graft-*Versus*-Host

“Unlike infection, Tx usually results in a double immune reaction: host-*versus*-graft and graft-*versus*-host.”

*-* Thomas E Strazl with the Nobel Laurate Rolf M Zinkernagel (36)

Whereas recipient-ICs undergo activation soon after transplant surgery, the graft-associated donor-ICs also change. These donor-ICs have unique surface antigens, which may be upregulated or altered due to activation by the diverse inflammatory state present after implantation that contribute to the immunodynamics of Tx. Several fundamental questions will be discussed regarding the migration and presentation of donor antigen-bearing ICs as well their components both within the transplanted organ and within the recipients’ secondary lymphoid organs. They are as follows:

### Do Allograft Leukocytes Migrate From the Inflamed Allograft to the Host and Vice Versa?

Based on skin graft experiments, Snell (76) pointed out that “intact cells from the graft may pass directly to the lymph nodes *via* the lymphatic vessels….this is an important factor in the development of the immune response. The evidence also suggests that donor lymphocytes in the graft play a particularly significant role *(p.450)”*. Donor ICs within the allograft are exposed to inflammation due to hypoxia and injury and are activated by proinflammatory cytokines (77). Strazl and others (36, 37, 77) demonstrated that leukocytes from the allograft, termed passenger leukocytes (PLs), may serve as the mediators for systemic graft *versus* host reactions as well as functioning as “primary immunogens” eliciting a recipient immune response.

The PLs may include pluripotent stem cells, cells of MO lineage such as MPs and DCs, and a variety of lymphocytes. Larsen et al. (38) documented that the donor-derived MHC alloantigen (HLA-II)-bearing DCs migrate out of mouse cardiac allografts into the recipients’ spleens and associate predominantly with CD4+ T lymphocytes. An increased number of leukocytes with the donor’s incompatible and unique antigens were detected in other tissues by about 2-weeks, and by 3-months in the circulation. This time frame coincided with the first appearance of *de novo* anti-allograft Abs. It is postulated that donor leukocytes may remain in the recipient *for many years* post-Tx (36, 37, 76, 77). Interestingly, the persistence of donor leukocytes is highest in liver and intestinal allografts and lowest in heart and kidney Tx (36, 37, 76–80). Evidently, migration of donor-ICs varies both with the organ type and severity of inflammation. Therefore, donor-ICs both within and outside the allograft require a closer examination to better understand the early events of the immunodynamics of Tx.

By using MHC-mismatched donor and recipient mice, it was noted that apparently donor-derived alveolar MPs are persistent for more than three and a half years in murine lung allograft recipients (81). These MPs expressing donor-associated biomarker**s** could initiate donor-specific immune responses by the recipient. This “persistent donor alveolar MP lineage” was identified as autofluorescent cells expressing CD45, CD11b, HLA-DR, the lectin CD169 (Siglec-1), the mannose receptor CD206, and the scavenger receptor CD163. They constitute about 7% of bronchoalveolar lavage cells. Transfer of these MPs to allogenic mice leads to the production of anti-HLA-I and anti-HLA-II IgG antibodies as well as autoantibodies (collagen V and Ka1-tubulin) (81). Similar alveolar MPs expressing donor markers on their surface are identified in human subjects (82).

Donor leukocytes may migrate relatively rapidly to recipient lymph nodes or spleen, wherein they present donor intact HLA directly to alloreactive T cells (83, 84). One major impediment is that the allograft lymphatic vessels, one of the routes of donor-PLs, are disconnected during surgical resection and their full reconnection with the recipient’s lymphatic vessels occur**s** 5 to 7 days after Tx. However, the alloreactive-T cell response has already been initiated by day 2 (85, 86). Additionally, in murine models, the allograft-DCs are targets of recipient NK and cytotoxic CD8+ T cells (87–89). The alloreactive recipient CD8+ T cells rapidly eliminate donor DCs from T-cell areas of draining lymph nodes through a perforin-dependent mechanism (88). Ly49D(+) CD127 (–) NK cells were recruited within draining lymph nodes and rapidly eliminated allogeneic H-2(d) DCs also through the perforin pathway (89).

**Figure 1:**
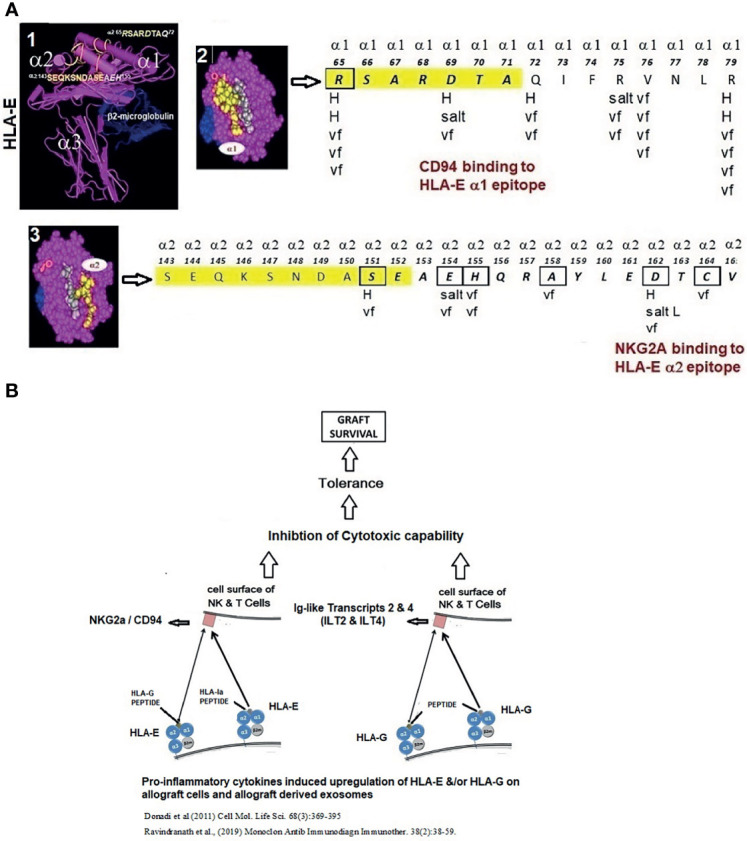
**(A)** Structure of HLA-E illustrating HLA-E specific epitopes on α1 and α2 helices. The interaction between the HLA-E amino acid sequences on α1 and α2 helices (boxes) and NKG2a and CD94 inhibitory receptors (arrows) involves H-bonding (H), van der Waal forces (vf), and salt linkages (salt) of the amino acids of HLA-E and the inhibitory receptors. **(B)** Proinflammatory cytokines upregulate not only HLA-E but also HLA-G. When HLA-E and HLA-G present non-allogenic peptides, they bind to the respective inhibitory receptors on the NK cells, leading to inhibition of cytotoxic capabilities of NK cells, promoting tolerance and graft survival.

In humans, however, this concern for rapid elimination of donor APCs may be somewhat abrogated. Human ECs and several leukocytes, including DCs, express HLA-E on their surface, which is upregulated by proinflammatory cytokines (90, 91). HLA-E primarily functions as the specific ligand for an inhibitory receptor (CD94/NKG2A) on NK and CD8+ T cells (92) (**Figure 1A**). Examining the effects of the presence or absence of HLA-E on the surface of CD94/NKG2A positive NK Cell line (LCL.221-AEH), inhibition of NK cell-mediated cytolysis by HLA-E was documented. Interestingly, CD94 and NKG2A bind to specific epitopes on the α1 and α2 heavy chains of HLA-E (92, 93) that are upregulated on endothelial cells under the influence of inflammatory cytokines. The HLA-E epitope-specific binding to CD94/NKG2A receptors on NK and CD8+ cells and the HLA-G binding Ig-like transcripts and inhibitory receptors on NK and CD8+ cells (**Figure 1B**) suggests that the non-classical HLAs generated following proinflammatory cytokine activation may protect PLs from the recipient’s NK/CD8+ T cell attack, representing a potent illustration of the interaction of inflammation with the immune response to Tx.

Strazl et al. (77) hypothesized that allograft acceptance may occur provided there are “responses of co-existing donor and recipient ICs, each to the other, causing reciprocal clonal expansion, followed by peripheral clonal deletion”. It is important to recall Mohankumar group’s (81) finding that cells carrying donor antigen are tolerated for more than three and a half years in murine lung allograft recipients. Although animal studies propose that such interactions could lead to tolerance, in humans the result could also be allograft rejection (37, 79, 81). This possibly depends on the state of inflammation in the microenvironment. Perhaps more important than infiltration of intact donor ICs may be uptake of donor subcellular fragments by recipient ICs.

### Do the Alloantigens Shed From the Inflamed Graft Get Adsorbed by the Cells of the Recipients?

Several *in vitro* studies have shown that DCs are capable of acquiring intact MHC molecules from other cells (other DCs, MPs, activated T cells, B cells, and tumor cells) (86–88, 94–100), representing the semidirect method of allorecognition. DCs can shed soluble HLA class I and class II molecules and costimulatory molecules that can be captured by other DCs (95–97). In experiments separating the donor and recipient DCs by 0.4 μm pore size membranes, Herrera et al. (99) demonstrated that direct contact between cells may not be necessary for the transfer of MHC molecules between cells. Furthermore, the control experiments done by direct contact showed enhanced transfer of intact HLA molecules between the cells. Indeed, donor HLA molecules were detected on the surface of the recipient’s APCs in the graft-draining lymphoid organs after murine heart and kidney Tx (83, 84).

The presence of donor-associated shed alloantigens detected on recipient APCs is facilitated by activation of pro-inflammatory factors. Such shed antigens enter into the circulation of the recipient post-Tx, even before the presumed migration of PLs. Furthermore, these antigens may be incorporated into the recipient’s ICs, and these recipient cells can be mistaken for migrating donor PLs. The transfer of antigens is not unidirectional but bi-directional, for that is how the graft associated cells acquire the recipient’s MHC molecules (96–98). *The relative ratios of alloantigens and autoantigens in the recipient’s as well as donor’s ICs may provide a better understanding of the outcome of a particular transplant (tolerance vs rejection). Further study is required to delineate these ratios.*

### Do Extracellular Vesicles (EVs), Including Exosomes, Being the Potential Carriers of Alloantigens From Donor Cells, Get Transferred to a Recipient’s Leukocytes?

EVs, including exosomes, also play a role in bi-directional antigen transfer. After fully mismatched skin or heart Tx in mice, extremely few or no donor passenger-DCs were detected in the recipient’s draining lymphoid organs (98). However, allograft-derived exosomes carrying donor MHC molecules were captured by the recipient-DCs, which present donor MHC molecules directly to alloreactive T cells (94). Peche et al. (95) demonstrated an effect of allogeneic exosomes on the modulation of immune responses *in vivo*, suggesting that, like donor cells, exosomes can stimulate or regulate antigen-specific immune responses including the promotion of tolerance. *Interestingly, it was the donor-derived but not syngeneic exosomes that induced a significant prolongation of allograft survival, with long-term graft survival in a few recipients.* During the first week after Tx, allografts from exosome-treated rats displayed a significant decrease in both graft-infiltrating leukocytes and the expression of proinflammatory IFN-γ mRNA compared with allografts from untreated animals. However, allogeneic donor-derived exosomes can also lead to increased anti-donor MHC class II alloantibody production promoting rejection (96). P*roinflammatory cytokines can reverse the tolerance that would have been promoted by donor ICs or donor cell-derived exosomes*, again demonstrating the interaction of inflammation with immunodynamics.

Marino et al. (100, 101) revisited the concept of antigen transfer from allograft to recipient leukocytes by exosomes derived from donor cells in allogeneic murine skin Tx. They could not find any evidence for the presence of donor PLs in the lymph nodes and spleen of the skin-grafted mice. However, they observed a high number of recipients’ leukocytes carrying allogeneic MHC molecules, acquired from donor exosomes. They demonstrated that *purified allogeneic exosomes induced proinflammatory alloimmune responses by T cells both in vitro and in vivo*, suggesting that the release of donor HLA-carrying exosomes could initiate recipient T-cell responses. The phenomenon of donor exosomes being taken up by the recipient’s APC for the presentation of donor-MHC on their cell surface is referred to as “allo-MHC cross-dressing” (95–104). The result is semidirect allorecognition by recipient ICs. As noted above, allogenic HLA molecules get recognized by T cell receptors *via* three distinct pathways;

- the direct pathway whereby recipient T-cells recognize *intact donor*-HLA molecules expressed on *donor* APCs,
- the indirect pathway involving *processed donor-*HLA molecules presented by *recipient*-MHC molecules expressed on *recipient* APCs, and
- a semi-direct pathway whereby *intact donor*-HLA molecules are expressed on *recipient* APCs *via* exosomal uptake (94–101).

Similarly, using a murine heart transplant model, Liu et al. (105) showed that the exosomes from donor-DCs, which migrated from the graft to lymphoid tissues, “cross-dressed” the recipient DCs. Exosomes were either internalized or remained attached to the recipient cDCs (cross-dressed DCs). Upon acquiring the exosomes, the recipient’s DCs became activated and triggered full activation of alloreactive T cells. Further, it was shown that a reduction in the number of recipient DCs after cardiac Tx drastically decreased the presentation of donor MHC to alloreactive T cells and delayed graft rejection in these mice.

Although the concept of exosome formation appears novel, the formation of EVs is known as “clasmatosis” and was reported in the literature on lymphocytes and phagocytes in vertebrates (106, 107) and hemocytes of invertebrates (108). These EVs were thought to pinch off from the plasma membrane (PM). However, studies on reticulocyte maturation lead to a more complex mode of formation of EVs (109). It was noted that small vesicles were formed by inward budding inside an intra-cellular endosome, leading to the formation of a multi-vesicular body (MVB), which could then fuse with the plasma membrane and release outside its internal vesicles (110). The word ‘exosomes’ was proposed for these EVs based on their size and endosomal origin (111), while other scientists reported that EVs could be categorized into two main classes: ectosomes and exosomes. Ectosomes are derived from direct budding off the plasma membrane and not from fusion with endosomes. Kowal and Tkach (111) have noted that the term “exosomes have been extensively used in the literature to refer to the totality of small EVs that sediment at high-speed ultracentrifugation (most commonly at 100,000 x g), even though the endosomal nature of the vesicles is poorly documented in most studies up to date (p.215)”.

We use the terms EVs and exosomes interchangeably, although the exact origin from endosomally derived MVBs is often not determined in the studies we cite, and thus it remains possible that what we refer to as exosomes may be ectosomes. The relevance, if any, of this distinction remains uncertain. EVs are composed of bi-layered lipid with embedded transmembrane proteins that enclose soluble proteins and nucleic acids. EVs emanate from all ICs, epithelial, and endothelial cells. They are also found in body fluids, particularly blood (112), semen (112–114), and urine (115).

The presence of EVs in multiple sites may be explained by the ability of EVs post-Tx to travel long distances to reach the recipient’s cells. A variety of *interaction mechanisms* may occur between EVs and recipient cells, such as immobilization on the recipient cell’s surface through specific receptors, signaling events, membrane fusion, endocytosis, or micropinocytosis (116–118). Some molecules that are involved in this interaction include ICAM-1, LFA-1, αv and β3 integrins, or tetraspanins CD9 and CD81. EVs release their content to recipient cells in various ways including acidic endo/lysosomes (111).

The protein profile of EVs appears to differ depending on both the origin of the cells and their physiological functions. EVs released by DCs contain HLA-I and HLA-II molecules (98, 99, 117, 118). Using immunoelectron microscopy, Zitvogel et al. (119) found that in human mature DCs (mDCs), multivesicular endosomes contain abundant HLA class-I molecules. MHC class I and II, CD63 and CD82, were also found in intraluminal 60-90 nm vesicles. EVs (60-90 nm), abundantly labeled with anti-MHC class I and II, CD63 and CD82 specific antibodies, were frequently observed at the outer side of the plasma membrane. These vesicles occur in mDC culture supernatants and were analyzed after isolating by differential ultracentrifugation. Over 90% of the homogeneous population of vesicles (60-90 nm diameter) were labeled with anti-CD63, anti-CD82, and anti-HLA-I and II antibodies. The MVBs in the luminal vesicles of immature DCs store HLA-II molecules. The vast majority of antigen-loaded HLA-IIs stably expressed at the plasma membrane by mDCs are synthesized after exposure to inflammatory stimuli (118). When these cells are activated, the luminal vesicles have the propensity to fuse with the plasma membrane, thus increasing the expression of HLA-II on the cell surface (118). These EVs containing HLA molecules are released under the influence of various cytokines, chemokines, and lipopolysaccharide (LPS). LPS-treated bone marrow DCs (BMDC) released EVs rich in HLA-II together with CD86 and ICAM-I (120, 121). IFN-γ-treatment of murine BMDCs also promoted an increase in CD80 (119). Similar results were observed with EVs released from IFN-γ treated DCs, which contained co-stimulatory molecules (CD40, CD80, and CD86) and ICAM-I (120, 122). EVs are also released from immature DCs (123). In the presence of cognate T cells, DCs secrete higher amounts of HLA-II-bearing EVs. When a peptide specifically recognized by T cells is added to a DC:T cell co-culture, there is an enhanced release of EVs carrying HLA-II (124). In addition to T-cells and DCs, B-cells may release EVs with important consequences. Confirming the endosomal EV secretion pathway in B cells, Raposo et al. (125–127) demonstrated the release of HLA-II-containing exosomes that were able to induce antigen-specific HLA class II-restricted T cell responses.

The presence of both mRNA and microRNA inside exosomes indicates that mRNA can be transmitted to another cell through EVs (128). Such transfer can occur from exosomes derived from the allograft ICs to the recipient’s ICs, suggesting a novel mechanism of cell-cell communication. The message passed on could result in either tolerance of the allograft or destructive inflammation leading to rejection. Donor ICs and recipient’s ICs can use EVs as means to exchange proteins, lipids, and nucleic acids.

The exact function of exosomes and other EVs and their defined role in the induction of inflammation is still unclear. Kowal et al. (111, 129) have extensively reviewed the molecular composition of DC-derived EVs, to illustrate the difference between EVs from DCs and other ICs and their clinical relevance. Their findings suggest that exosomes will be a cornerstone for the understanding of the immunodynamics of inflammation, including elucidation of their secretory pathways within ICs, their identification in body fluids, and the discovery of their nucleic acid (RNA) content. Hence, EVs are major players in the immunodynamics of Tx and *are intimately related to the inflammatory milieu.*

Monitoring exosomes in the blood of patients might be a promising noninvasive method to evaluate the status of allografts (130). Thus, the immunodynamics of transfer of donor HLA to the recipient’s antigen-presenting cells is critically important to understand. In a recent review on exosomes, Gonzalez-Nolasco et al. (17) summarize that “donor exosomes rather than passenger leukocytes are the main source of antigens for allorecognition by T cells after Tx. However, this remains to be demonstrated as well as the contribution of exosomes and antigen cross-dressing in rejection and tolerance of allografts (page 25).”

### Functional Implications of Allograft Derived “Passenger Leukocytes”, EVs, MHC or Non-MHC Antigens

It is evident that allograft ICs, their EVs, and cell membrane MHC and/or non-MHC antigens get into the recipient’s microenvironment, particularly into the lymphatic channels and nodes. The result could be rejection or tolerance. Immunological tolerance is considered as an antigen-induced failure of the immune response, brought about by either inactivation (131–134) or elimination (135) of the immunocompetent cells, but only for specific donor antigens. Indeed, Hilgert (136) has experimentally documented that the antigen cross-dressing from donor-derived cell membrane-associated MHC contribute to the tolerance of an allograft.

During skin grafting of monozygotic and dizygotic twins, Medawar et al. (131–134) observed that skin grafts from dizygotic twins was not rejected identically to monozygotic twins. Spleen cells from foreign inbred strains were inoculated into mouse embryos and testing skin grafting in their adult life with the skin of the original donor of the spleen cells, and the grafts were not rejected. Argyris (137) further validated the above findings by rendering the newborn C3H mice tolerant to CBA skin homografts by neonatal injection of CBA spleen cells. The offspring of these tolerant C3H mice were more susceptible to a tolerance-inducing stimulus from the CBA spleen cells than were the offspring of untreated C3H mice. Medawar (134) proposed that donor antigen-exposed immunocompetent alloreactive ICs (T cells) are inactivated to recognize the antigen-bearing allografts. In contrast to the above hypothesis, Burnet (135) proposed that the donor cells or their antigens reach the thymus of the recipient resulting in deletion (as opposed to inactivation) of clones of the immunocompetent alloreactive cells.

## Consequences of Early Inflammation: Activation of Immune Cells and ECs

Two simultaneous events are evident in the early phases of Tx:

(1) The migration of EVs and ICs carrying graft-associated antigens into the recipient’s lymphatic and circulatory system, and
(2) Entry of the recipients’ ICs into the allograft.

The former event may possibly suppress the immunocompetent cells of the recipient and promote tolerance, whereas the later event involving infiltration of the recipients’ mononuclear cells into the inflamed graft tissues, potentially resulting in allograft rejection. These events mimic accumulation and activation of lymphocytes, primarily CD4+ T helper cells and CD8+ T suppressor cells, due to inflammation in various lung diseases such as idiopathic pulmonary fibrosis, sarcoidosis, and hypersensitivity pneuomonitis (138–141).

**Figure 2:**
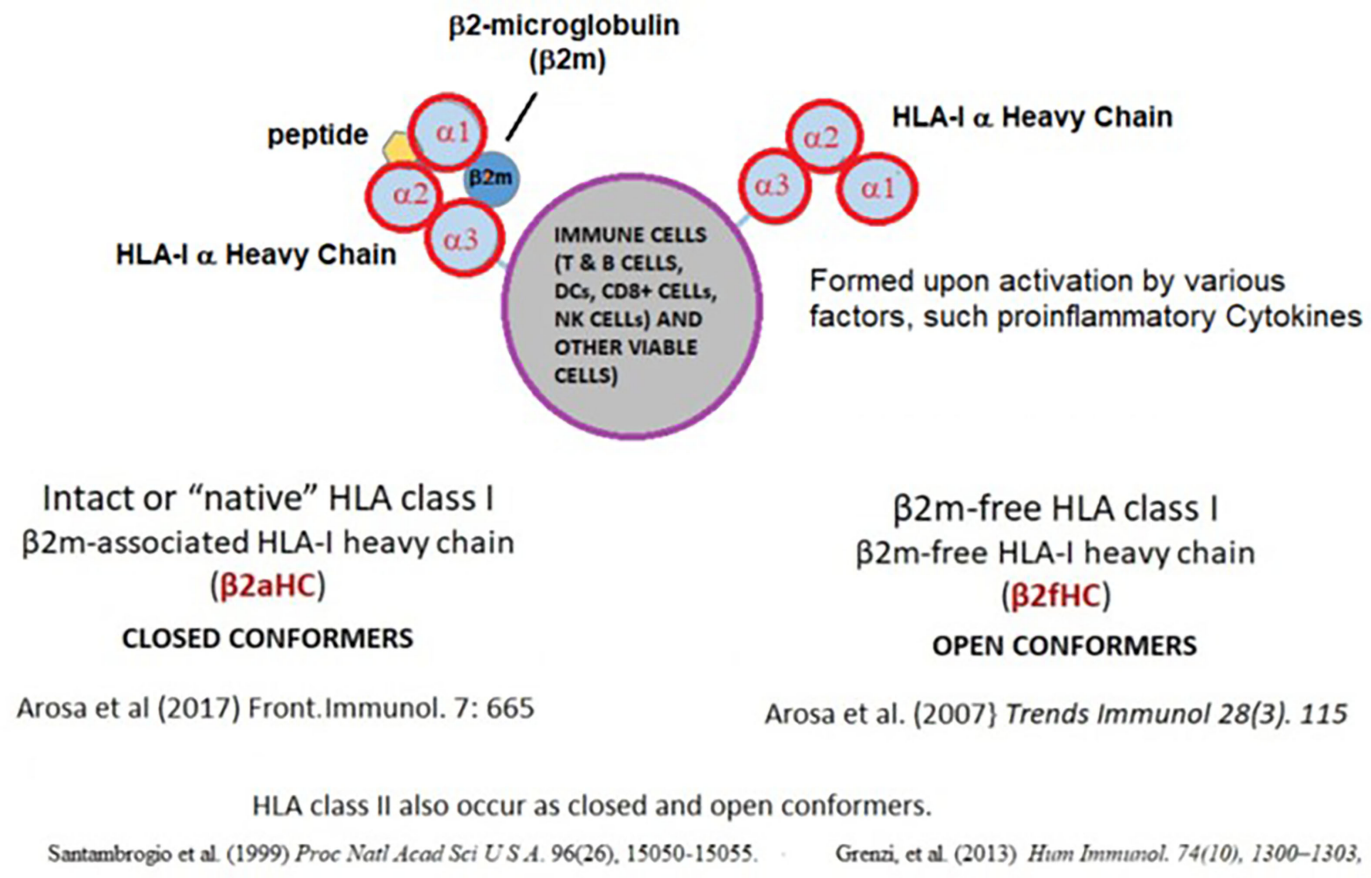
Impact of pro-inflammatory cytokines on HLA-I molecules. On the cell surface, HLA class-I occur as dimers for HLA-I (α-chain and β2-microglobulin (β2m) complexed with a short peptide, designated as Closed Conformers (CCs). Upon activation by pro-inflammatory factors, many ICs express monomeric variant of the HLA, called “Open Conformers (OCs). Commercial microbeads coated with HLA CCs admixed with OCs will not provide accurate assessment of serum antibodies against intact cell surface HLA.

The most striking event following *in vivo* or *in vitro* activation of ICs is the overexpression of cell surface HLA molecules, which occur as trimers, composed of α-chain and β2-microglobulin (β2m) and peptide for HLA-I and α- and β-chains and a peptide for HLA-II. Such trimers are designated as “Closed Conformers (CCs)” (142). However, upon activation by pro-inflammatory factors, many ICs, express monomeric α-Heavy Chains (HC), called “Open Conformers (OCs) (143) (**Figure 2**). HLA-OCs are not “denatured” HLA heavy chains (HC), but “naturally-occurring” HLA-HCs. Several reports (27–32) document their expression on the surface of metabolically activated cells, including human T-lymphocytes activated *in vitro* and *in vivo*, as well as by EBV-transformed B-cells, CD19+ B-cells, *ex-vivo* CD8+ T cells, CD56+ NK-cells, CD14+ monocytes, extravillous trophoblasts and MOs, B-cell lines (RAJI, NALM6), and a myeloid cell line (KG-1A). The kinetics of conformational alterations in the naturally occurring β2m-free glycosylated HLA-I OCs after activation were investigated in healthy human T-cells (40). DCs also express HLA-I and HLA-II OCs, with the former being capable of cross-presenting antigens after endocytosis (96, 125, 144). The elongated cytoplasmic tail of naturally occurring HLA-I OCs is tyrosine phosphorylated and play a role in signal transduction (39).

Inflammation in the allograft microenvironment leads to the activation of both recipient lymphocytes and donor cells, particularly ECs. This activation enables shedding of their surface antigens, both non-MHC molecules and OCs of classical and non-classical MHC (32, 93, 145–149), which results in the exposure of cryptic epitopes of these antigens. Interestingly, some of the shed antigens (e.g. HLA-G isomers) could promote tolerance, possibly mediated by the inhibition of cellular immune functions (148 –149). However, the presence of HLA-II on activated T-helper cells simultaneously with the activation of B lymphocytes (96, 125, 144) led to the production of *de novo* anti-allograft Abs. Production of anti-allograft Abs commence after the advent of tolerance. Activated host CD8+ suppressor cells eliminated donor leukocytes residing in the allograft and created an altered cytokine profile that resulted in loss of tolerance and subsequent Abs production.

## Impact of Inflammation-Activated ECS on the Allograft

ECs play a significant role in upregulating inflammation soon after Tx. ECs line the inner lumina of vessels of the lymphatic and blood vascular systems. Also, smooth muscle cells and pericytes provide support for the vascular structures. ECs exhibit organ-specific adaptations in shape and function (150). ECs in the central nervous system form the blood-brain barrier (151), in the uterus express estrogen receptors (152) in the high endothelial venules of lymph nodes express Fas ligands (153), and in the endocardium fold up and adapt to the constant heartbeat. ECs of the immune system function not only as a transport device for mobile ICs, but also secrete chemokines, interleukins, interferons, and growth factors. They enable recruitment of ICs and regulate leukocyte extravasation at the specific sites of inflammation by inducible expression of adhesion molecules like E-selectin, P-selectin, ICAM, or VCAM (154). Coordination of ECs of different organs with the inflammation-induced immune responses of the organ post-Tx deserves critical study.

### Storage Organelles and Immune Receptors of the Endothelium

ECs possess rod-shaped storage organelles called Weibel-Palade bodies (WPBs) (155), which contain multiple pre-made, pro-inflammatory, and pro-hemostatic proteins, including the leukocyte receptor P-selectin, the pro-hemostatic glycoprotein von Willebrand factor (VWF), and pro-inflammatory cytokines. In some ECs, WPBs upregulate IL-8 (156, 157) and angiopoietin-2 (158) after endothelial activation and are released by exocytosis (159, 160). These contents of WPBs enter the blood and initiate hemostasis and promote inflammation and leukocyte recruitment (161, 162).

At the site of tissue injury during surgery, activated ECs release WPBs with the following results: P-selectin recruits leukocytes to protect the wound (163): IL-8 (164) and IL-6 (165) direct the course of inflammation, endothelin-1 causes vasoconstriction to close off the affected area (166), angiopoietin-2 destabilizes endothelial junctions and their barrier function for flexibility during tissue repair (167), and tissue plasminogen activator prevents excessive fibrin formation (168).

ECs also express several innate immune receptors including the toll-like receptor (TLR) family that recognize pathogen-associated molecular patterns (PAMPs) (169–171). ECs express all the members of this family, which include TLR1, TLR2, TLR3, TLR4, TLR5, TLR6, and TLR9 (172). In normal resting ECs, TLR7, TLR8, and TLR10 are not observed; however, they are induced upon inflammation. Upon ligand binding, TLRs on ECs are dimerized and activated to signal NF-kappa B and MAPK resulting in pro-inflammatory cellular responses. These responses include an increase in vascular permeability, production of inflammatory cytokines, presentation of adhesion molecules to recruit leukocytes, and the switch to a procoagulant state. *Specifically, direct activation of TLR1/2, TLR3, and TLR4 elicit a strong pro-inflammatory response by stimulating the production of cytokines such as IL-6, IL-8, TNF-alpha, and IL-1 beta* resulting in altered adhesion molecule expression, (E-selectin, P-selectin, ICAM, and VCAM), elevated vascular permeability through reduced junction protein claudin-5, and induced secretion of several procoagulant factors (173). Elevated blood and tissue sugar levels induce an inflammatory stimulus for the activation of ECs, which is also mediated *via* TLR2 and TLR4, leading to shedding of the glycocalyx of the endothelium (174). This enables improved leukocyte adhesion and increased reactive oxygen species (ROS) production. EC- glycocalyx shedding is executed by heparinase, matrix metalloproteinases (MMPs), and ROS. A range of cell adhesion receptors on ECs mediates the capture, rolling, arrest, and crawling of leukocytes on the luminal endothelial cell surface. This is the prelude for the actual transmigration of lymphocytes and other leukocytes through the endothelial barrier, known as the diapedesis process.

### Structural and Functional Heterogeneity of the Endothelium

Endothelial phenotypes display heterogeneity in structure and function in both health and disease (175), reflecting diverse functional requirements of specific body tissues. For example, the endothelial layer of renal arteries and veins is nonfenestrated and continuous, whereas the endothelium of glomerular and peritubular capillaries is fenestrated to promote increased filtration and transendothelial transport (176, 177). The expression of HLA class II antigens is quite high on quiescent glomerular and peritubular microvascular ECs (178, 179), in contrast to the presence of only HLA class I antigens on the quiescent endothelium of different vascular beds. Quiescent glomerular ECs express very low levels of the angiotensin II type 1 receptor (AT1R), whereas the endothelium of preglomerular vessels expresses the angiotensin II type 2 receptor (AT2R) (180, 181). Paradoxically, most of the *in vitro* clinical researches on ECs was done on human umbilical vein ECs (HUVECs) or aortic macrovascular endothelium, but rarely on ECs derived from the kidney micro- or macrovascular systems. The structure and function of the ECs may not only differ among the quiescent vasculatures of different organs but the ECs may change remarkably depending on their exposure to diverse inflammatory mediators.

ECs of an allograft, such as that of a kidney allograft, are never quiescent and are subjected to constant pressure from inflammatory stimuli. In such situations, activation of ECs upregulates all classes of HLA and non-HLA antigens including angiotensin receptors, which occurs pre-implantation during brain death and the organ retrieval process. Such activation may lead to endothelial dysfunction, as is known to occur during ischemia and reperfusion injury, and can be augmented with immunosuppressive therapeutic drugs (182). There is an imminent need to critically look into the effects of other immunotherapeutic agents administered before and soon after transplant surgery on endothelial function.

### Endothelial Chimerism in Allografts: Does It Render Protection From Immune Attack?

After kidney Tx, the ECs of the vasculature and glomerular and periglomerular capillaries of the allograft are activated due to inflammatory stimuli and express antigen profiles different from that of the native kidneys of the recipient. Tolerance of an allograft could depend on how soon the allograft makes changes on the structure and antigenic profiles of the ECs to the native (recipients) state, although this is not straightforward. Jooste et al. (183–186), observed two major differences when rat skin grafts were exposed to anti-graft sera. Some grafts succumbed to treatment with antigraft sera. However, some grafts survived the treatment. The resistance to antiserum was attributed to the replacement of the graft endothelium by the recipient’s cells. In humans, the presence of ECs of recipient origin has been documented in allografts (187, 188). Sinclair (189) performed sex chromatin counts on the ECs of 40 human kidneys transplanted to recipients of the opposite sex. The donor endothelium persisted in all except in three severely damaged grafts. In these three patients, a high proportion of the ECs in peritubular capillaries and veins were derived from the host, suggesting that endothelial chimerization may occur *after* injury to the donor endothelium and thus is not required to achieve tolerance.

Similarly, examining kidney grafts using immunohistochemistry for MHC-I antigens, ABO-blood-group antigens, and *in-situ* hybridization for X and Y chromosomes, Lagaaij et al. (190) provided evidence to show that part of the ECs in the blood vessels of a transplanted donor kidney expressed the MHC antigens of the recipient. A strong correlation between the percentage of recipient ECs in the peritubular capillaries and the type of graft rejection (*r*=0·71, p<0·0001). Recipient cells were present mainly in grafts of patients who had rejection, especially among patients with vascular rejection (191, 192). In grafts of patients without rejection, only sporadic recipient ECs were detectable. These observations again indicate that such replacement may be the result of allograft injury. No recipient’s cells could be observed in the allograft of patients without rejection. It is postulated that the allograft endothelium damaged by vascular rejection is repaired by the recipient’s ECs, thereby supporting the concept of endothelial chimerism of donor and recipient as a response to injury. *These results stand quite opposite to Medawar’s hypothesis that graft adaptation may occur when a transplant gradually becomes less immunogenic and resistant to rejection due to gradual replacement of the ECs of the donor by those of the recipient*. The term endothelial chimerism was used for the partial replacement of donor ECs in an allograft by the ECs of the recipient or “the presence of recipient derived ECs in the donor organ.”

Lagaaij et al. (190), comparing the pre-and post-transplant histology of HLA-A Ab-stained biopsies from eight kidney transplant recipients, observed that a part of the cells in the endothelial lining of the vessels of a transplanted kidney expressed the HLA class-I antigens of the recipients, while the other part of the endothelial lining expressed the HLA class-I of the donors. One of the donor kidneys was HLA-A3 negative and it was transplanted into an HLA-A3-positive patient. After immunostaining at 6 months post-Tx, a portion of the cells of the endothelial lining showed positivity for the recipient HLA type (HLA-A3) indicating chimerism. In the same biopsies, infiltration of leukocytes was observed in the region of the native endothelium. When examining the biopsies of other patients, more than 30% of the endothelium were positive for recipient antigens. In some biopsies, the tubules *per se* were positive only for donor HLA, while the graft-infiltrating leukocytes in the vessels were positive for recipient HLA. It is not clear whether antigen cross-dressing occurs between leukocytes and ECs, although the concept of exosomes detailed above strongly supports such a possibility.

Rienstra et al. (193), studied the nature of donor and recipient ECs in a rat model of renal Tx and observed endothelial chimerism in capillaries (glomerular and peritubular) but not in arterioles and arteries. In humans, endothelial chimerism was predominant in the peritubular capillaries. Both in animal models and humans, chimerism may lead to vasculopathy and it has been suggested that endothelial chimerism may be a consequence of inherent damage to the endothelium during graft placement. Furthermore, endothelial chimerism may signify a functional repair process mediated by recipient cells to maintain endothelial integrity.

Interestingly, van Poelgeest et al. (194) recorded a higher incidence of chimerism in female recipients (8/8) over male recipients (8/16). Donor graft EC replacement occurred *earlier post-Tx in females* than in male recipients. They hypothesized a putative role for VEGF in endothelial cell turnover and possible stimulation of VEGF under the influence of estrogens in females. *Interestingly, the long-term outcome from a larger cohort of human and animal* (195, 196) *renal allograft recipients are reported to be better in female than in male recipients, supporting Medawar’s hypothesis.*

All of the above studies raise the following question: Do the faster and earlier replacement of the endothelial lining by the recipient’s endothelium under inflammation preserve allograft function? More research is clearly needed.

Vascular muscle cells underlying ECs are also inflammatory mediators. The wall of the vascular system of an allograft, whether it is kidney, liver, or heart, consists of tunica intima which consists of a layer of luminal ECs supported by a basement membrane, and an underlying stroma containing layers of vascular smooth muscle cells. When the allograft tissues become hypoxic upon surgical injury, not only the ECs but the vascular smooth muscle cells are activated to elicit proinflammatory mediators, including *superoxide-initiated inflammation that results in the production of potential proinflammatory cytokines (IL-1, IL-6) and chemokines (CCL2, and CXCL10) by both types of* cells (197).

## Inflammation Biomarkers: Dynamic Role of IL-6

Although graft-associated cells, EVs, and/or soluble antigens migrate into the recipients’ lymphatic system to alter the activities of the recipient’s immuno-competent cells, in most human Txs, these events fail to promote tolerance. The failure could be due to (1): the inflammation induced cyto- and chemokines (2), the allograft’s failure to generate more cells and their derivatives (EVs, antigens) (3), the intensity of preoperative or post-surgical inflammation in the microenvironment of the allograft (4), the immunosuppressive therapies, and/or (5) the immunosuppressive properties HLA-polyreactive antibodies generated by HLA OCs (93, 198–200).

Inflammation-induced cytokines could be a major impediment for tolerance and the primary determining factor for allograft rejection. Chakraborty and Sarwal (201) have extensively reviewed cyto- and chemokine biomarkers in Kidney Tx. Karxzewski et al. (202) summarized cytokines and chemokines associated with acute rejection (IFNγ, CXCL-9, CXCL-10), acute ABMR (CXCL-10, GM-CSF), acute TCMR (CXCL-9, CXCL-10, GM-CSF), and chronic rejection (IL-4, IL-6, IL-10). It appears that the preoperative inflammatory state of a patient about to undergo Tx defines the consequences of the early immune events of Tx. In critically ill patients, as well as with ICU and overall length of stay in the hospital, high levels of IL-6, IL-8 and IL-10, and CCL-2 are associated with mechanical ventilation (203, 204). Sonker et al. (205) reported that the patients undergoing allograft rejection displayed significant increases in circulating IL-6. Jordan et al. (206–208) observed in renal-Tx patients that abnormal production of IL-6 created multiple pathogenic responses including chronic inflammation, immune stimulation, and neo-vascularization, and evaluated anti-IL6 receptor therapy to improve long-term graft survival in chronic antibody mediated rejection in phase I and II clinical trials. We now focus on the dynamics and regulation of IL-6 in transplantation as a prototypical inflammatory cytokine upregulated post-Tx.

### Nature of IL-6 and Its Presence in the Allograft Microenvironment

**Figure 3:**
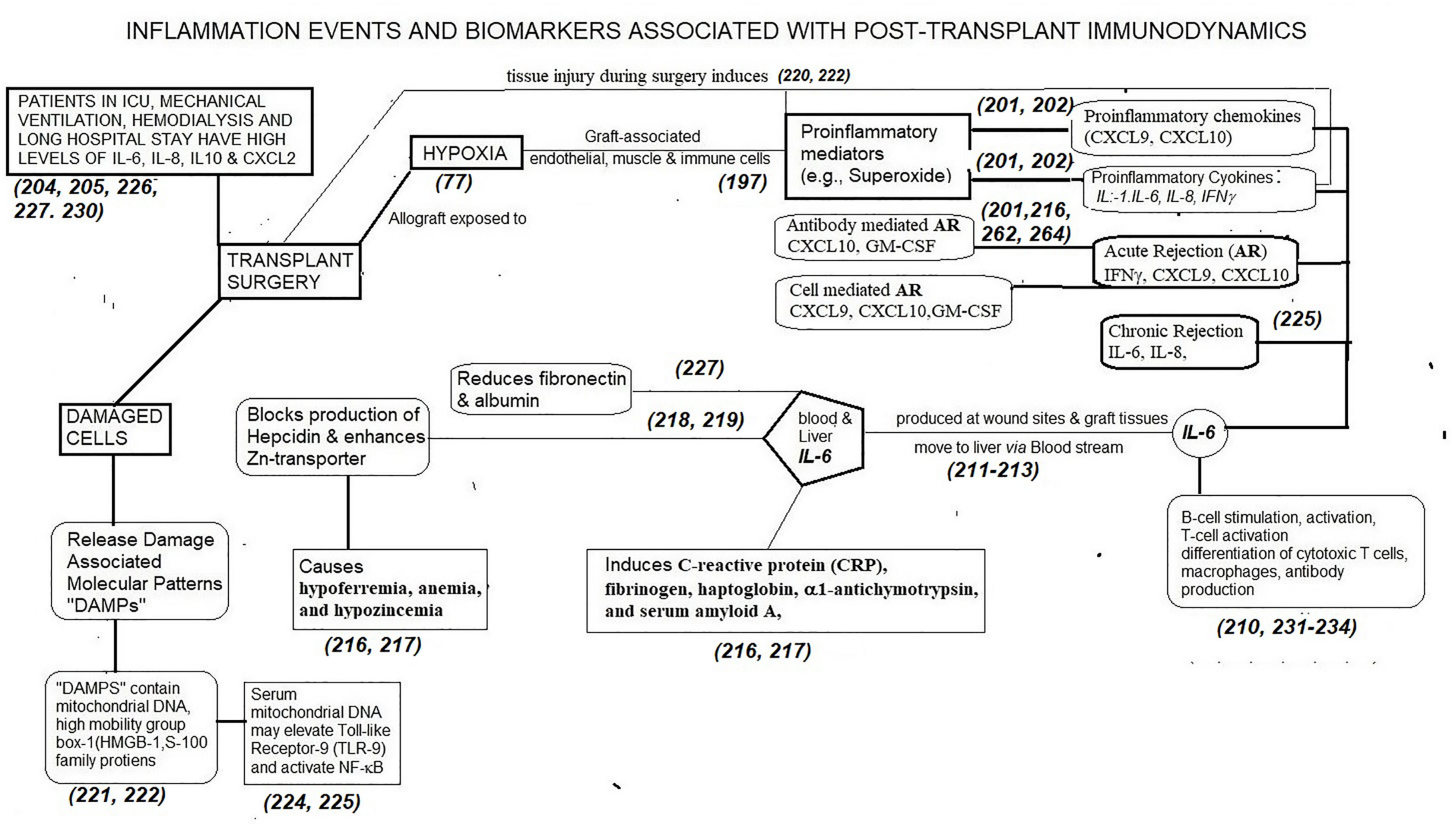
The dynamic role of proinflammatory mediators generated following transplant surgery and the role of one of the cytokines (IL-6) in generating biomarkers post-Tx. Other cytokines and chemokines also generate such biomarkers. There is an imminent need to identify time-based production of these inflammation biomarkers post-Tx to prevent allograft rejection, to promote tolerance of allografts and to develop appropriate personalized, dynamic, chemo-immunotherapeutic strategies.

IL-6 was initially described as interferon β2, hepatocyte stimulating factor, cytotoxic T-cell differentiation factor, B-cell differentiation factor, or B-cell stimulatory factor-2 (209–212). These descriptions of IL-6 signify the multiple potential immunoregulatory actions. The core protein of IL-6 is 20 kDa, with glycosylation accounting for the size of 21–26 kDa (209–211). IL-6 is produced by all APCs, mesenchymal cells, ECs, fibroblasts, and many other cells in response to diverse stimuli. IL-6 is involved in the acute phase response, B cell maturation, and MP differentiation. The primary roles of IL-6 are activation of lymphocytes and induction of *de novo* HLA-monospecific and HLA-polyreactive Ab-formation against closed and open conformers of MHC and non-MHC antigens. Of the various cytokines and biomarkers, IL-6 has a significant role both preoperatively and e*arly* post*-*Tx. **Figure 3** narrates the inflammation events and biomarkers, primarily IL-6, associated with post-transplantation immunodynamics.

Surgery, brain death, cold storage, and reperfusion are potent inducers of IL-6 (209–213). Allograft-ECs, smooth muscle cells as well as the recipient ICs infiltrating the allograft elicit IL-6 production. At the initial stage of inflammation at a wound site, IL-6 is synthesized and moves to the liver through the bloodstream, followed by the rapid induction of an extensive range of acute-phase proteins, such as CRP, fibrinogen, haptoglobin, α1-antichymotrypsin, and serum amyloid A, along with a reduction of fibronectin, and albumin (211–217). Persistently high level of serum amyloid-A generated by IL-6 can result in serious complications of chronic inflammation such as amyloidosis (216). IL-6 is also involved in both blocking the production of hepcidin and enhancing zinc transporter levels, contributing to hypoferremia, anemia, and hypozincemia (217, 218).

Augmentation of serum IL- 6 levels precedes the elevation of body temperature and serum acute-phase protein concentrations after surgery (219). Damaged cells or dying cells under non-infectious conditions such as sterile surgical operations, release “Damage-associated molecular patterns (DAMPs)” which directly or indirectly promote inflammation. DAMPs contain a variety of molecules such as mitochondrial (mt) DNA, high mobility group box 1 (HMGB1), and S100-family of proteins (220–222). Serum mtDNA levels in trauma patients are thousands of times higher than in controls and this elevation leads to TLR9 stimulation and NF-kB activation (222, 223).

Enhanced IL-6 mRNA synthesis by peripheral blood mononuclear cells is observed in patients with chronic renal failure (223, 224) and on hemodialysis (225). IL-6 is an independent predictor of mortality in incident dialysis patients (225–227). Higher pretransplant IL-6, IL-8, and CCL-2 levels were correlated with post-transplant primary graft dysfunction in lung Tx (228). Verleden et al. (229) noted that high IL-6 and IL-8 levels in broncho-alveolar lavage within 24-hrs of lung-Tx in 336 patients were significantly associated with prolonged length of stay in the ICU, delayed hospital discharge, and an increased prevalence of grade 3 primary graft dysfunction.

The approximate concentration of the serum-IL-6 in a healthy individual is 1 picogram/ml (10^7^ molecules/ml). Bologa et al. (226) observed several-fold increase in hemodialysis patients, consequently resulting in hypoalbuminemia and hypocholesterolemia. IL-6 was the strongest predictor of mortality in univariate and multivariate analysis. Extending the above findings, Pecoits-Filho et al. (227) showed that the predictive value of elevated circulating IL-6 levels was similar in patients starting peritoneal dialysis.

IL-6 produced by APCs can modulate specific differentiation of naïve CD4+ T cells into effector Th1 or Th2 cells following Tx (210, 230). IL-6 also induces the differentiation of CD8+ T cells into CTLs (231). It is anticipated that the concentration of IL-6 within the allograft could be much higher than in the serum/plasma soon after surgery. IL-6 can induce the differentiation of activated B cells into Ab-producing plasma cells (232, 233). Over-synthesis of IL-6 results in hypergammaglobulinemia and autoantibody production. Most importantly, IL-6 can function within the vascular endothelium of the allograft as well as outside in its microenvironment.

Hence, IL-6 links innate immunity to the acquired immune response (210–213). Th1 cells produce primarily interferon-gamma (IFNγ) and tumor necrosis factor (TNF), which are prerequisites for cell-mediated inflammatory reactions. Th2 cells secrete interleukin IL-4, IL-5, IL-10, and IL-13, which mediate B cell activation and antibody production. IL-6 produced by APCs can shift the Th1/Th2 balance toward the Th2 direction, by promoting and Th2 differentiation and inhibiting IFNγ production and Th1 differentiation (230, 231). This shift to a Th2 profile may result in alloantibody production.

Waiser et al. (234) and Van Oers et al. (235) have studied IL-8 expression in serum and urine after renal TX. Rejections within 2 months of renal Tx were accompanied by elevated serum-IL-6 concentrations (17 +/- 4.8 pg/ml, P < 0.05) and urine-IL-6 (114 +/- 27 pg/ml, P < 0.005), compared to controls. The values returned to normalcy (0-5 pg/ml) after successful treatment. The urine-IL-6 was higher (93%) than serum-IL-6 (54%). The specificity in serum (70%) and urine (60%) was reduced by infection, acute tubular necrosis, and anti-thymocyte globulin treatment. In biopsy tissue, IL-6 and IL-6R were both elevated during rejection. Especially, mononuclear cells within the interstitial infiltrate stained positive. However, the amount of IL-6 positive cells did not correlate with peripheral IL-6 concentrations.

The IL-6 effects in the allograft microenvironment can be enumerated as follows:

1) activation of any cell that expresses IL-6 receptor (IL-6R) (236–238) 2); activation of Th2 cytokine production in CD4+ T lymphocytes *via* the transcription factor C/EBP (239) 3); increased generation of Th17 cells together with TGFβ (241) 4); suppression of CD4+ T-regs by inhibiting their differentiation and hence increasing the Th17/T-reg ratio (240, 241); and 5), activation, maturation and proliferation of naïve B cells to plasma cells leading to the production of high-affinity Abs (232, 233). These changes enhance the chance for ABMR. Indeed, IL-6 is the major cytokine involved in Ab-mediated transplant vasculopathy and graft loss.

### Dynamics and Regulation of IL-6 and IL-6 Receptor During Early Phases of Transplantation

Understanding the dynamics and regulation of IL-6 signaling is critical to elucidate the physiological and pathophysiological functions of IL-6 and to formulate novel therapeutic strategies. IL-6 binds to a specific receptor (IL-6R), an 80 kDa type I transmembrane protein (236, 237, 242). IL-6-IL-6R complex associates with a second transmembrane protein, gp130 (242–245). This glycoprotein is expressed by all cells in the body, and it serves as both a signal transducer and a common receptor unit of IL-6 and the IL-6 type cytokine family (242–247). Upon IL-6 binding to IL-6R, membrane-bound gp130 (mgp130) dimerizes to initiate a variety of intracellular signaling pathways (244, 246, 247). The membrane-bound IL-6R primarily occurs on hepatocytes, neutrophils, monocytes, and CD4+ T-cells.

A soluble IL-6R (sIL-6R) is generated by proteolytic cleavage of the membrane-bound IL-R and the sIL-6R can bind to IL-6 (248–250). In humans, sIL-6R can be generated by the translation of an alternatively spliced mRNA (249). The IL-6 signaling *via* the membrane-bound IL-6R is called ‘classic signaling’ and the IL-6 signaling *via* the sIL-6R is termed called ‘trans-signaling’ (237, 244, 245). During inflammation, IL-6 binds to sIL-6R (249–252). The IL-6-sIL-6R complex binds to membrane-bound gp(mgp)130 with higher affinity than IL-6-membrane bound IL-6R (253). However, soluble gp130 (sgp130) also exists (253–255), and it will also bind the IL-6-sIL-R complex with higher affinity than mgp130 and neutralize it. Consequently, the high amounts of sIL-6R and sgp130 in the blood constitute a buffer for IL-6. Since the concentration of sgp130 exceeds the concentration of sIL-6R, the sIL-6R concentration may limit the signaling activities of circulating IL-6 (252–254).

In obese individuals, IL-6 trans-signaling is involved in the infiltration of MPs into the adipose tissue, which leads to a chronic inflammatory state (256). Blockade of IL-6 trans-signaling with sgp130Fc completely prevented MP infiltration (257). MP infiltration in adipose tissue is the strongest predictor of insulin resistance in obese individuals (258). Thus, the pro-inflammatory activities of IL-6 may be mainly mediated by the trans-signaling mechanism.

### Regulation of IL-6 and IL-6 Receptor During the Late Phases of Transplantation

The transplant-associated early upregulation of cytokines and inflammatory biomarkers should not be misconstrued with either cytokine upregulation occurring during infection or that persisting months after Tx. Such persisting or late upregulation may result from silent or chronic viral and bacterial infections, autoimmune diseases, cardiovascular complications, and injuries. For example, it is known that the genome of Human Herpes Virus 8 (HHV8) encodes a protein that shows 25% identity with human IL-6 (259). The “viral IL-6” possesses the ability to stimulate gp130 in an IL-6R-independent manner, and this viral protein can activate far more target cells than human IL-6 (260). Some of these inflammatory factors may also lead to tubular damage in renal allograft recipients and vasculitis in other organ allografts (261). Repeated measurements of critical inflammation biomarkers associated with Tx, namely CRP, IL-6, and TNF-α, early after kidney Tx found that in the absence of rejection, these inflammation markers may immediately increase but are followed by a decrease to baseline levels within a week (262–265). Subsequent increases of these inflammatory markers in allograft recipients may depend on the received therapies and other inflammatory stimuli.

To recognize the major role of IL-6 in the initiation and production of anti-allograft Abs, attempts were made to prevent IL-6 interaction with its receptor (IL-6R) on CD4+ T cells and B-cells. These strategies were initiated before Tx. Two anti-IL-6R Abs are approved by the US-FDA: Tocilizumab, a humanized monoclonal Ab (Acetemra^®^TCZ, Genentech), and Siltuximab, another monoclonal Ab (Sylnl^®^Janssen Biotech). Tocilizumab treatment in patients with inflammatory autoimmune diseases increased peripheral T-regs significantly (266–269). Phase I/II trials of tocilizumab as a desensitizing agent for HLA-sensitized patients were reported (207–209, 266, 269). The *de novo* appearance or increasing levels of circulatory inflammation biomarkers, such as soluble CD30, that correlate positively with the loss of a graft, are valuable for monitoring the course of the anti-IL-6 therapy. Patients treated with tocilizumab developed weight gain and increased levels of triglycerides and cholesterol, showing that caution is needed regarding duration and dose (269). This is critical when considering tocilizumab as a replacement, or addition to, more standard desensitization with IVIg plus rituximab. Above all, Grabbers et al. (228) caution that blockade of a single cytokine can be desirable at the site of inflammation but could be devastating at off-target sites. They point out that the “future therapeutic strategies should therefore take into consideration that cytokines act in complex networks and should be inhibited locally rather than systemically and if possible even not on all cells at inflammatory sites” (p. 94).

Not all allograft recipients with HLA mismatches produce HLA-DSA (270). Class et al. (271) proposed the theory of differential immunogenicity of HLA mismatches, which proposes that the failure of HLA mismatches to induce effector T lymphocytes or antibody production could be due to individual genetic factors. Based on this theory, Martin et al. (272) tested the hypothesis that genetic factors may be responsible for the tendency of some renal allograft recipients to produce DSA. They examined the influence of single nucleotide polymorphisms (SNP) arising in immune regulatory genes, particularly the influence of SNPs on the IL-6 gene upon the presence or absence of DSA production in the recipient post-Tx. A statistically significant association was observed for the recipient IL-6 rs1800795 SNP and the production of anti-HLA DSA post-Tx. In the antibody-positive group (*n*= 29), 97% of the recipients carried the GG or GC genotype compared with 77% in the antibody-negative group (*n* = 66) (*P* = 0.02), suggesting the presence of G at this position is associated with increased DSA. This finding correlates with an *in vitro* study on patients with systemic-onset juvenile chronic arthritis (273), which showed the rs1800795 GG genotype to be a high IL-6 producer phenotype. A statistically significant association was also observed for the donor IL-6 rs1800795 SNP and the production of HLA-DQ-specific DSA in allograft recipients.

All the above studies raise the following question: Can modulation of factors such as IL-6 or other inflammatory mediators be therapeutically altered to tip the balance of the alloimmune response from rejection to tolerance?

## Conclusion

The primary objective of this review is to understand the role of inflammation in the immunodynamics of transplantation with the hope of differentiating tolerance-promoting inflammation from that enhancing allograft rejection, so that potential therapeutic modifications can be achieved. The first and foremost aspect of inflammation and inflammatory biomarkers in Tx-patients is the ESD of the patient waiting for an organ.

The review emphasizes the need to first consider the factors that may influence pretransplant inflammation and inflammatory biomarkers in the potential recipient. These include the specific organ that has failed, age, sex (parous or non-parous, if female), HLA class I and II profiles, prior infections, and comorbidities, diabetes, hypertension, and autoimmune/inflammatory diseases. Also, prior immunosuppressive and chemotherapeutic drug exposure should be considered. Clearly, research is needed to determine if a detailed profile including the levels of circulatory and urinary chemo- and cytokines and their membrane-bound and soluble receptors (eg., IL-6 and IL-6R) should be obtained before surgery and monitored after surgery, concomitant with an in-depth profile of circulating resting *versus* activated CD4+/CD8-, CD4-/CD8+, CD19+/CD5+ and CD19+CD5- B cells, CD56+ NK cells, T-reg cells and monocytes. A profile of monomeric HLA molecules on the ICs also deserves consideration since they may be acquired by DCs or shed leading to production of HLA-polyreactive antibodies. These profiles may vary with the nature of the organ that has failed.

After Tx, allograft antigens, either as intact cells (PLs), EVs such as exosomes, or as shed antigens, migrate to the lymph nodes and lymphoid organs of the recipient *via* the lymphatic vessels, representing the foremost event in allorecognition. These alloantigens may be incorporated onto recipient APCs through “cross-dressing”. Notably, the result could be either allograft tolerance or rejection. Proinflammatory biomarkers may indicate the direction of the immune response and may contribute to the outcome. Therefore, continuous monitoring of the aforementioned biomarker profiles may be necessary soon after surgery, and this also requires extensive research. Such monitoring hopefully would enable a more precise immunomodulation to avoid both under- and over- immunosuppression. The ultimate goal is personalized immunoregulatory therapy to enable tolerance of the allograft.

There is a also need to examine the impact of therapeutic agents before and soon after Tx on EC function and dysfunction. Tolerance of an allograft may depend upon how soon the allograft changes the structure and antigenic profiles of the ECs relative to the recipient. Studies on the benefit of endothelial chimerization have been conflicting. A high proportion of ECs in peritubular capillaries and veins of a kidney allografts may be derived from the recipient. Endothelial chimerization is found to be higher in female recipients than in males along with longer graft survival in the former. However, such chimerization may be the result of endothelial injury, and not the cause. For example, in grafts of patients without rejection, only sporadically recipient ECs were detectable. Clearly, here too more research is needed.

In conclusion, the data we have outlined indicate that inflammation and inflammatory biomarkers play a prominent role in determining the balance between tolerance and rejection. As these areas are better understood, perhaps personalized specific anti-inflammatory therapy may become available for transplant recipients. (10,633 words/30 pages).

## Author Contributions

MR developed the hypothesis, objective and wrote the main frame of the article, prepared tables and figures. FH wrote inflammation related aspects and provided new and significant insights, focused on references and cross checking the references. EF contributed to writing of the manuscript and discussed pros and cons of the findings and added new information. All authors contributed to the article and approved the submitted version.

## Funding

Professor Mark Terasaki, son of Late Professor Dr. Paul Ichiro Terasaki, provided financial support for this and related original research cited in the review. The funder was not involved in the study design, collection, analysis, interpretation of data, the writing of this article or the decision to submit it for publication.

## Conflict of Interest

The authors declare that the research was conducted in the absence of any commercial or financial relationships that could be construed as a potential conflict of interest.

## Publisher’s Note

All claims expressed in this article are solely those of the authors and do not necessarily represent those of their affiliated organizations, or those of the publisher, the editors and the reviewers. Any product that may be evaluated in this article, or claim that may be made by its manufacturer, is not guaranteed or endorsed by the publisher.
